# Low expression of circulating microRNA-328 is associated with poor prognosis in patients with acute myeloid leukemia

**DOI:** 10.1186/s13000-015-0345-6

**Published:** 2015-07-17

**Authors:** Li Liu, Ren’an Chen, Yangping Zhang, Wen Fan, Fang Xiao, Xueqian Yan

**Affiliations:** Department of Haematology, Tangdu Hospital, Fourth Military Medical University, No.1, Xinsi Road, Xi’an, Shaanxi 710038 People’s Republic of China

**Keywords:** Circulating, miR-328, Acute myeloid leukemia, Prognosis

## Abstract

**Background:**

Dysregulation of circulating miR-328 has been identified in several tumors and is associated with prognosis of patients. However, the expression pattern of miR-328 and the impact on prognosis has not yet been studied in acute myeloid leukemia (AML). The purpose of this study is to investigate the expression status of miR-328 and its clinical significance in AML patients.

**Methods:**

RNA was extracted from plasma of 176 patients with newly diagnosed AML and 70 healthy volunteers. The miR-328 expression was examined by Realtime quantitative PCR. The association of circulating miR-328 expression with clinicopathological factors and prognosis of AML patients was statistically analyzed.

**Results:**

The expression of miR-328 was significantly downregulated in AML patients (median value 22.99, range: 3.63-242.0) compared with those of healthy controls (median value 89.17, range: 12.05-397.7; *P* < 0.001), and miR-328 expression was markedly increased in patients after treatment than before (23.40 ± 1.76 vs. 46.61 ± 3.83, *P* < 0.001). Moreover, low levels of miR-328 were associated with a higher white blood cell count and BM blast count (*P* = 0.026 and *P* = 0.003, respectively), and lower hemoglobin and platelet count (*P* = 0.004 and *P* = 0.022, respectively). Patients with low miR-328 expression had a relatively poor overall survival (*P* = 0.022) and shorter relapse-free survival (*P* = 0.008) than those with high miR-328 expression. In addition, low miR-328 expression was an independent prognostic factors for both OS (*P* = 0.017) and RFS (*P* = 0.023).

**Conclusions:**

Circulating miR-328 downregulation is a common event and is associated with poor clinical outcome in AML patients.

## Background

Acute myeloid leukemia (AML), the most common type of acute leukemia in adults, is a clonal disorder caused an accumulation and differentiation arrest of myeloid blasts in the bone marrow and blood. The pathologic mechanism of AML can be largely explained by cytogenetic aberrations, acquired mutations and dysregulated gene expression [1, 2]. Based on cytogenetic information, AML patients are classified into three risk-based categories: favorable, intermediate, and poor, with a 5-year overall survival (OS) rate of 55 %, 24 %-42 %, and 11 %, respectively [3]. Treatment of AML has dramatically improved over the past several decades, with improvements in risk assessment, post-remission chemotherapy and hematopoietic stem-cell transplantation. However, the cause of AML is not yet fully understood. Therefore, early and accurate diagnosis of AML is essential for optimal treatment outcome and may deeply improve the prognosis of patients with AML.

MicroRNAs (miRNAs) are a class of non-coding small RNAs of ~22 nucleotides that regulate expression of target genes at the post-transcriptional level [4]. MicroRNAs function by directly binding to their potential target site in the 3’untranslated region (3’UTRs) of specific target mRNAs, resulting in the repression of mRNA translation or the degradation of target mRNAs [5]. Since the discovery of the first miRNAs, these small genes have added a new layer of complexity to the regulation of normal and pathological cell functions. Recent studies have indicated a key role of miRNAs in biological processes including cell proliferation, differentiation, apoptosis, as well as cancers and cardiovascular diseases [5, 6]. Currently, aberrant expression of miRNAs appears to be a common characteristic of hematological malignancies, including leukemias [7, 8]. Dysregulation of single miRNAs such as miR-212 [9], miR-124-1 [10], miR-181 [11] and let-7a-3 [12] has been found to be associated with the outcome of AML patients.

Recently, it has been reported that miRNAs are present in serum or plasma in a stable and reproducible fashion, and the unique expression patterns of serum or plasma miRNAs can be used as a new class of effective biomarkers for various diseases [13–15]. MiR-328, known as a tumor suppressor, is involved in the cancer development and progression [16, 17]. MiR-328 was reported down-regulated in chronic myelogenous leukemia blasts and glioblastoma tissues. However, a previous report found that peripheral blood miR-328 expression was up-regulated in non-small cell lung cancer (NSCLC) patients [18]. Wang et al. found that plasma miR-328 concentrations were significantly elevated in acute myocardial infarction (AMI) patients compared to those control subjects [19].

However, to the best of our knowledge, no previous reports exist concerning the expression status of circulating miR-328, the prognostic value and the role of this miRNA in AML. Thus, the aim of the present study was to investigate the correlation of circulating miR-328 with clinicopathological features as well as the prognosis of the patients with AML. Our findings may provide the better understanding on the roles and its clinic implications of circulating miR-328 in the development and progression of AML.

## Methods

### Patients and follow-up

From February 2010 to September 2014, 176 newly diagnosed de novo AML patients from the Department of Hematology at Tangdu Hospital of Fourth Military Medical University were enrolled in this study; there were 86 males and 90 females, with a medium age of 39.7 (range 16.2–67.6) years. 70 unrelated healthy adult donors were collected as controls; all the control subjects were matched with patient population in terms of age and sex. None of these controls had previously been diagnosed with any type of malignancy or other benign disease. AML patients were diagnosed according to standard diagnostic methods including cytomorphological, cytochemical, immunological and cytogenetic evaluation. The diagnosis and classification of AML patients were based on the French-American-British (FAB) and World Health Organization (WHO) criteria combined to immunophenotyping and cytogenetic analysis [20–23]. 124 patients received standard induction chemotherapy consisted of 1 or 2 courses of daunorubicin (45 mg/m^2^ daily for 3 days) combined with cytarabine (100 mg/m^2^) by a 7-day continuous intravenous infusion. AML complete remission (CR) was defined as a normocellular BM containing less than 5 % blasts and showing evidence of normal maturation of other marrow elements; a neutrophil count of 1 × 10^9^/L and a platelet count of 100 × 10^9^/L. 76 patients achieved CR, and then given high- or medium dose cytarabine-based chemotherapy for consolidation according to their physical condition. Patients were followed up for a median 26 months (range 5–51 months); Patients without death or relapse by the time of last follow-up were censored on that date. Overall survival (OS) was defined as the time from the diagnosis of AML to any cause of death. Relapse-free survival (RFS) was defined as the time between the achievement of complete remission and the time of the hematological relapse or death. This study was approved by the Ethics Committee Board of Tangdu Hospital of Fourth Military Medical University. Informed consent was obtained from each participant according to the committee’s regulations. Details of clinical characteristics of the patients are provided in Table 1.

**Table 1:** Clinicopathological variables of 176 patients with newly diagnosed AML and expression of miR-328

Clinicopathological variables	Cases (176, n/%)	miR-328 expression		*P* value
		low (125)	high (51)	
Age (years)				
≤60	126 (71.6)	90 (72 %)	36 (70.6 %)	0.997
>60	50 (28.4)	35 (28 %)	15 (29.4 %)	
Gender				
Male	86 (48.9)	60 (48 %)	26 (51.0 %)	0.847
Female	90 (51.1)	65 (52 %)	25 (49.0 %)	
WBC (×10^9^/L)				
<10	73 (41.5)	53 (42.4 %)	30 (58.9 %)	0.026
≥10	103 (58.5)	82 (57.6 %)	21 (41.1 %)	
HGB (g/L)				
<80	110 (62.5)	87 (69.6 %)	23 (45.1 %)	0.004
≥80	66 (37.5)	38 (30.4 %)	28 (54.9 %)	
PLT (×10^9^/L)				
<50	91 (51.7)	72 (57.6 %)	19 (34.2 %)	0.022
≥50	85 (48.3)	53 (42.4 %)	32 (62.8 %)	
Blast in BM				
<50 %	79 (44.9)	45 (36 %)	34 (66.7 %)	0.003
≥50 %	97 (55.1)	80 (64 %)	17 (33.3 %)	
FAB subtype				
M1	35 (19.9)	25 (20 %)	10 (19.6 %)	0.909
M2	44 (25)	32 (22.6 %)	12 (23.5 %)	
M3	11 (6.2)	7 (5.6 %)	4 (7.8 %)	
M4	26 (14.8)	20 (16 %)	6 (11.8 %)	
M5	60 (34.1)	41 (32.8 %)	19 (37.3 %)	
WHO Classification				
AML with t(8;21)	19 (10.8)	15 (12 %)	4 (7.8 %)	0.074
APL with t(15;17)	24 (13.6)	12 (9.6 %)	12 (23.5 %)	
AML without maturation	15 (8.5)	8 (6.4 %)	7 (13.7 %)	
AML with maturation	39 (22.2)	30 (24 %)	9 (17.6 %)	
Acute myelomonocytic leukemia	26 (14.8)	21 (16.8 %)	5 (9.8 %)	
Acute monoblastic and monocytic leukemia	53 (30.1)	39 (31.2 %)	14 (23.6 %)	
Karyotype classification				
Favorable	72 (40.9)	56 (44.8 %)	16 (31.4 %)	0.570
Intermediate	83 (47.2)	55 (44 %)	28 (51.9 %)	
Unfavorable	21 (11.9)	14 (11.2 %)	7 (13.7 %)	
Complete Remission				
Yes	76 (43.2)	55 (44 %)	21 (41.1 %)	0.861
No	100 (56.8)	70 (56 %)	30 (58.9 %)	

Note: *WBC* white blood cell, *HGB* hemoglobin, *PLT* platelet, *FAB* French–American–British, *WHO* World Health Organization

### Plasma collection and RNA extraction

Blood samples were collected in EDTA-K_2_ tubes and processed within 1 h of collection. Cell and nucleic acids free plasma was isolated from all blood samples using a 2-step centrifugation protocol (3000 g for 10 min and 12000 g for 5 min, all at 4 °C). The supernatant was transferred to RNase/DNase free tubes and stored at −80 °C. The plasma was first spiked with miScript miRNA mimic SV40 (Qiagen, Hilden, Germany, 2 μM, 1 μl per 100 μl plasma). Total RNA was isolated from the plasma using TRI reagent BD (MRC, USA) according to the manufacturer’s instructions and dissolved in 20 μl of RNase-free water. RNA sample concentration was quantified by NanoDrop ND-2000 (Thermo Fisher Scientific, USA). Quality of RNA was generally checked by the ratios of A260/A280 and A260/A230 and RNA integrity was assessed by electrophoresis through denaturing agarose gels.

### qRT-PCR analysis of plasma miR-328

Total RNA (1 μg) from each sample were converted into cDNA using PrimeScript RT reagent kit with gDNA Eraser (TaKaRa, Japan) and miRNA-specific stem-loop RT primer or SV-40 primers (Applied Biosystems, USA). Briefly, the reverse transcription reaction was performed in 20 μL mixture containing 10 μL of genomic DNA elimination reaction solution, 4 μL 5 × PrimeScript Buffer, 1 μL PrimeScript RT Enzyme Mix, 1 μL stem-loop RT primer or SV-40 primers, and 2 μL RNase Free water. For synthesis of cDNA, the reaction mixture was incubated at 42°C for 15 min, 85°C for 5 s, and then held at 4 °C. Quantitative reverse transcriptase polymerase chain reaction (qRT-PCR) was performed on ABI 7500 fast real-time PCR system (Applied Biosystems, USA) using SYBR Premix *Ex Taq* TM II (TaKaRa, Japan) according to the manufacturer’s instructions. PCR program conditions were 95 °C for 30 s, followed by 40 cycles of 95 °C for 3 s and 60 °C for 30 s. A melting program was performed after each reaction to validate the specificity of the expected PCR product. The Ct values greater than 36 were considered as not expressed. Resultant miRNA levels were normalized using spiked-in SV40. The relative expression level of miR-328 was calculated by the equation of 2^-ΔCt^ (ΔC_t_ = C_t miR-328_ - C_t__spiked-in SV40_) [24]. The fold changes in miR-328 were calculated using the 2^-ΔΔCt^ method [25]. Each sample was analyzed in triplicate and the mean expression level was calculated.

### Statistical analysis

Statistical analysis was performed with SPSS 16.0 for Windows (SPSS, Chicago, IL). Continuous data are presented as mean ± SD or median with interquartile range. Categorical variables are presented as counts and percentage. The Mann–Whitney U-test was used to evaluate the significant difference of expression of miR-328 between the AML patients and healthy controls. The paired *t* test was used to evaluate the difference expression of miR-328 before and after chemotherapy. Chi-square analysis or Fisher exact test was performed to evaluate the difference of categorical variables. Univariate logistic regression analyses for the association with the risk of survival and relapse to AML were tested first for miR-328 expression, age, gender and other clinical characteristics, and those factors were included into a second multivariate logistic analysis. Survival curves were plotted using the Kaplan-Meier method, and differences were tested using the log-rank test. Differences were considered to be statistical significant when P value was less than 0.05.

## Results

### MiR-328 was downregulated in AML patients

**Fig. 1:**
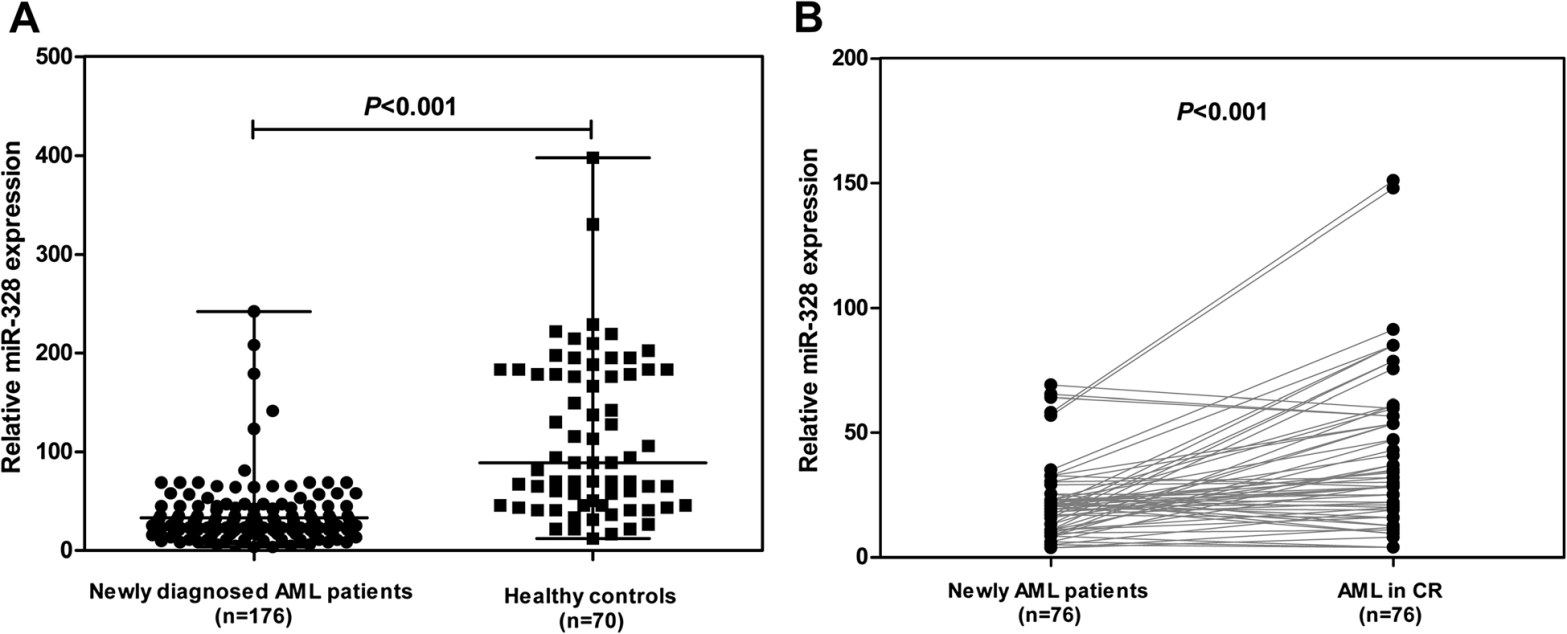
Circulating miR-328 expression in newly diagnosed AML patients and healthy controls detected by quantitative real-time polymerase chain reaction (qRT-PCR) analysis. **a** Expression levels of miR-328 in newly diagnosed AML patients (median expression value 22.99, range: 3.63-242.0; n = 176) and healthy controls (median expression value 89.17, range: 12.05-397.7; n = 70). Data shown are the Median and range, each symbol represents an individual AML patient and healthy controls, horizontal lines indicate median values and range. Mann–Whitney U-test and associated P value is indicated. **b** Expression levels of miR-328 in 76 AML patients before (mean value 23.40) and after complete remission (CR) ( mean value 46.61). Each symbol represents an individual AML patient at diagnosis and at achievement of CR, paired *t* test and associated *P* value is indicated

The miR-328 expression levels were detected in plasma samples from patients with AML and healthy controls by qRT-PCR. As shown in Fig. 1a, plasma concentration of miR-328 was markedly downregulated in AML patients (median expression value 22.99, range: 3.63-242.0) relative to those in healthy controls (median expression value 89.17, range: 12.05-397.7; *P* < 0.001). In addition, 76 patients who achieved CR were monitored for miR-328 during the course of treatment. The mean fold change of miR-328 in these AML patients was markedly increased when CR was achieved after chemotherapy (mean expression value 23.40 ± 1.76 vs. 46.61 ± 3.83, *P* < 0.001).

### Correlations between the levels of miR-328 and the clinicopathological factors in AML patients

To identify the clinical relevance of miR-328 expression in AML patients, correlations between miR-328 expression and clinicopathological parameters were made. AML patients expressing miR-328 at levels less than the mean expression level (33.1) were assigned to the low expression group (mean expression value 20.87, n = 125), and those samples with expression above the mean value were assigned to the high expression group (mean expression value 63.03, n = 51). As shown in Table 1, low levels of miR-328 were associated with a higher white blood cell count and BM blast count (*P* = 0.026 and *P* = 0.003, respectively), and lower hemoglobin and platelet count (*P* = 0.004 and *P* = 0.022, respectively). However, other clinical characteristics, including age (*P* = 0.997), gender (*P* = 0.847), FAB subtype (*P* = 0.909), WHO classification (*P* = 0.074) and karyotype classification (*P* = 0.570) were not directly related to the low level of miR-328.

### Association between miR-328 expression and clinical outcomes of AML patients

**Fig. 2:**
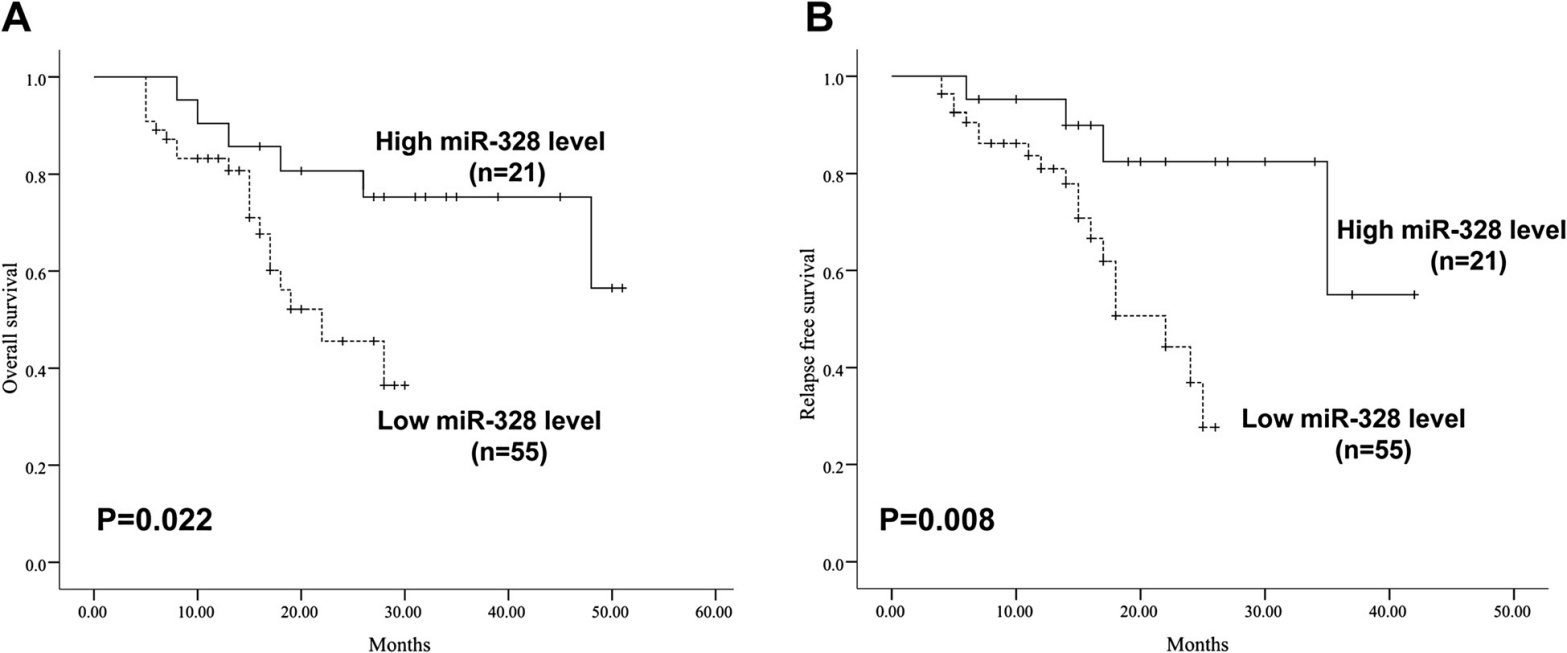
Kaplan-Meier survival curves for overall survival (OS) and relapse-free survival (RFS) according to circulating miR-328 expression from 76 AML patients in complete remission (CR). **a** The OS rate of AML patients in CR with high or low miR-328 expression; **b** The RFS rate of AML patients in CR with high or low miR-328 expression. The *P*-value was calculated using the log-rank test

To investigate the prognostic impact of miR-328 low expression in AML, survival analysis was performed in 176 cases. There were no differences in the OS and RFS between two groups (*P* = 0.137 and *P* = 0.339, data not shown). Among 176 cases, 124 patients received standard induction chemotherapy, The CR rate after two cycles of chemotherapy was 44.0 % (55/125) in the low-expression group, compared with 41.2 % (21/51) in the high-expression group (*P* = 0.861), there was no significant difference between the two groups. Moreover, the OS of 124 AML patients with high miR-328 expression was shorter than those with low expression, but the difference was not statistically significant (*P* = 0.176). However, among those obtained CR, overall survival curves and relapse-free survival curves in high-miR-328 group (n = 21) and low-miR-328 group (n = 55) are shown in Fig. 2. Patients with low mR-328 expression have shown significantly poorer overall survival (*P* = 0.022, Fig. 2a) and shorter relapse-free survival (*P* = 0.008, Fig. 2b) than those with high miR-328 expression.

Univariate analyses showed that higher white blood cell count (*P* = 0.004), lower hemoglobin (*P* = 0.009), platelet count (*P* = 0.017), BM blast count (*P* = 0.012) and miR-328 level (*P* = 0.009) were significantly associated with OS (Table 2), while higher white blood cell count (*P* = 0.009), lower hemoglobin (*P* = 0.04) and lower miR-328 level (*P* = 0.01) were found to be prognostic factors for RFS (Table 2). Furthermore, the multivariate Cox regression analysis revealed that low miR-328 expression was an independent prognostic factors for both OS (Hazard ratio [HR] =2.67; 95 % confidence interval [CI], 1.12-4.73; *P* = 0.017) and RFS (HR = 1.914; 95 % CI, 1.01-3.27; *P* = 0.023) of AML patients. Statistical values of the expression of miR-328 and other clinical parameters derived from Cox stepwise proportional hazards model were indicated in Table 2.

**Table 2:** Univariate and multivariate analyses of factors associated with survival and relapse of AML patients

Factors	OS				RFS			
	Univariate	Multivariate			Univariate	Multivariate		
	*P*	Hazard Ratio	95 % CI	*P*	*P*	Hazard Ratio	95 % CI	*P*
Age, years (≤60 *vs* >60)	0.741	N.A.	N.A.	N.A.	0.675	N.A.	N.A.	N.A.
Gender (male *vs* female)	0.821	N.A.	N.A.	N.A.	0.514	N.A.	N.A.	N.A.
WBC (<10 *vs* ≥10, ×10^9^/L)	0.004	1.37	0.67-2.04	0.04	0.009	1.39	0.81-2.33	0.039
HGB (<80 *vs* ≥80, g/L )	0.009	1.25	0.66-1.79	0.076	0.040	1.120	0.82-1.63	0.247
PLT (<50 *vs* ≥50, ×10^9^/L)	0.017	1.64	1.07-2.87	0.038	0.274	N.A.	N.A.	N.A.
Blast in BM (<50 % *vs* ≥50 %)	0.012	1.93	1.09-3.21	0.041	0.213	N.A.	N.A.	N.A.
miR-328 expression (high *vs* low )	0.009	2.67	1.12-4.73	0.017	0.01	1.914	1.01-3.27	0.023

Abbreviations: *WBC* white blood cell, *HGB* hemoglobin, *PLT*, platelet

## Discussion

Nowadays, it is becoming evident that expression patterns of microRNAs appears to be a common characteristic of hematological malignancies including leukemias, some of them can be a valuable tool for the diagnosis and prognosis of human cancer [26, 8]. Recently, it has been reported that microRNAs are circulating in serum/plasma. Additionally, microRNAs, such as miR-134 [19], miR-218 [27, 28], miR150 and miR-324 [29] in human serum or plasma have been shown to have much stronger stability than high molecular weight RNA due to their resistance to RNase digestion [15]. These findings make microRNAs a potentially non-invasive tools for cancer diagnosis using blood samples [15].

The present study has confirmed, for the first time, that the plasma miR-328 may serve as useful diagnostic and prognostic biomarkers for patients with AML. MiR-328 has been suggested to be a tumor suppressor by targeting proto-oncogene serine/threonine-protein kinase PIM1 and translational regulator protein hnRNP E2 [26]. Eiring et al. reported that miR-328 was down-regulated in chronic myelogenous leukemia blasts, and low expression of miR-328 in CML is associated with progression to the blast crisis phase of the disease [16]. Wu et al. observed that miR-328 expression is decreased in high-grade gliomas and is associated with worse survival in primary glioblastoma [17]. However, miR-328 was also expressed at high levels in several cancers. Ulivi et al. reported that circulating miR-328 expression was significantly higher in non-small cell lung cancer (NSCLC) patients than in healthy donors [18]. Wang et al. found that plasma miR-328 concentrations were significantly elevated in acute myocardial infarction (AMI) patients compared to those control subjects [19]. In our research, plasma concentration of miR-328 was markedly downregulated in patients with newly diagnosed AML compared with healthy controls. Moreover, the expression of miR-328 was significantly elevated after chemotherapy when patients achieved CR, suggesting that expression of miR-328 is consistent with tumor burden. Our results were consistent with other studies regarding CML and glioblastoma [17, 16], indicating that miR-328 plays an essential role in the original and/or progression of AML.

MiR-328 is proposed as a suppressor gene because its expression is decreased in several types of cancers and mediates proliferation, invasion and metastasis of cancer cells. It is demonstrated that enforced expression of miR-328 could remarkably attenuate glioma cell proliferation, invasion and migration [30]. MiR-328 could also inhibit epithelial-mesenchymal transition (EMT) via targeting CD44 [31]. These findings indicate that miR-328 plays a direct role in the modulation of cancer progression and may be useful as a novel prognostic or progression marker for cancer.

In the current study, we found that downregulatation of miR-328 in AML patients was significantly associated with a higher WBC count and blast count in BM, and lower HGB and PLT counts, which represented more aggressive clinicopathological features. In addition, AML patients with low miR-328 expression tend to have poorer OS and RFS than those with high miR-328 expression, indicating that expression of miR-328 has an important value in AML prognosis classification. In a logistic regression analysis, an association was observed between miR-328 expression and the risk of both survival and relapse of AML patients. It was observed that those patients with low expression of miR-328, presented a high risk of OS (*P* = 0.009) and RFS (*P* = 0.017) to AML compared to those patients who had high expression of miR-328 expression. In addition, multivariate analyses performed showed that miR-328 low expression is an independent predictor for OS (HR = 2.67, 95 % CI, 1.12-4.73; *P* = 0.017) and RFS (HR = 1.914, 95 % CI, 1.01-3.27; *P* = 0.023) of AML patients, which was in agreement with recent findings in glioblastoma [17]. Taken together, our results suggest that circulating miR-328 maybe function as a suppressor gene in the development of AML, and may have an adverse effect on prognosis in apart of AML patients.

## Conclusion

In summary, our study offers the evidence for the first time that circulating miR-328 is downregualted in AML patients, and lower miR-328 level is closely associated with distinct clinical and biologic characteristics in AML patients. Furthermore, lower miR-328 level is an independent poor prognostic factor for OS and RFS. However, the precise molecular mechanisms by which miR-328 is downregulated in AML need to be further investigation.
